# Coping With the Emotional Impact of Working in Cancer Care: The Importance of Team Working and Collective Processing

**DOI:** 10.3389/fpsyg.2022.877938

**Published:** 2022-07-15

**Authors:** Pádraig Cotter, Anneka Holden, Caroline Johnson, Sarah Noakes, Catherine Urch, Alex King

**Affiliations:** ^1^Department of Clinical Health Psychology, Clarence Wing, Imperial College Healthcare NHS Trust, St Mary’s Hospital, London, United Kingdom; ^2^Research Society of Process Oriented Psychology United Kingdom, London, United Kingdom; ^3^Department of Surgery and Cancer, Imperial College London, South Kensington Campus, London, United Kingdom; ^4^Department of Surgery, Cardiovascular and Cancer, Imperial College Healthcare NHS Trust, St Mary’s Hospital, London, United Kingdom

**Keywords:** collective processing, cancer care, integrative, transference, mirror neurons, Polyvagal Theory, process-oriented psychology, systemic

## Abstract

Hospitals provide the vast majority of cancer care. A necessary focus on survival has meant that they are less well-developed in terms of supporting patients with the emotional impact of cancer; and in supporting the frontline staff who contend with this. An integration of psychotherapeutic and neurobiological findings is used to develop an understanding of the patient-staff relationship and impact of high levels of distress within it. This includes reference to Transference and Countertransference, Mirror Neurons and Poly Vagal Theory. This paper considers how patients can unconsciously “transfer” emotional distress on to healthcare practitioners; and how this evokes an emotional response from the practitioner via the mirror neuron system (MNS). This can allow the practitioner to “feel into” the patient’s experience and develop a more nuanced understanding. However, it may also activate emotions connected to the practitioner’s life and can leave them feeling overwhelmed. The practitioner’s capacity to regulate their own emotional arousal, via the vagus nerve, has a significant impact on their ability to support the patient and themselves within emotionally distressing interactions. This dynamic often unfolds without either party having significant awareness of it. A Systemic and Process-Oriented perspective is taken to understand this within the broader context of a hospital-based structure; and consider how practitioners on frontline teams may or may not support each other in working collectively with high levels of distress. A team’s level of understanding and attunement to emotional experiences as well their primary relational and communication style has significant bearing on capacity for emotion-and-relationship focused coping. A failure to work with the emotional and relational interconnection between patients and staff can contribute to isolated patients, disconnected staff, conflict within teams and an overarching system lacking in compassion. However, due to the often unconscious nature of such processes and limited understanding or training on them, they are regularly left unaddressed. Over time, this can have an accumulated effect on everyone. Group-based collective processing is considered in terms of how it can be used in supporting practitioners to integrate an emotional and relational way of working with a problem-focused approach and integrated into regular daily working.

## Introduction

Modern healthcare has become increasingly focused on data, outcomes, measurable results, and cost-effectiveness (Perdelle et al., 2020). Many related advances and challenges are the by-product of a particular way of perceiving and experiencing the world. This philosophical perspective or epistemology known as Positivism has guided much of Western society (Economics, Education, Politics, and Healthcare) over the past 200 years (Brown, 2021). The scientific method and medical model are heavily grounded within it (Wilson, 2000). This viewpoint holds that there is an objective reality that can be quantified and measured. A related perspective that has had a major impact on modern healthcare is Cartesian Dualism, more commonly referred to as the mind-body split (Short, 2020).

The benefits of objectivist medical science in terms of the advancement of healthcare have been numerous (Ahn et al., 2006). This approach to dissecting and learning about different parts of the human body has guided specialized approaches to treating disease and pathology (Federoff and Gostin, 2017). It has given rise to the development of highly evolved departments within hospitals (e.g., gastro, dermatology, urology) and as a result, healthcare systems are very organized, structured and uniform.

Hospital-based systems have many advantages because of how their development has been informed by positivism, the scientific method and the medical model. The corollary of this is that these systems often marginalize more relativistic, phenomenological, and holistic perspectives (Miles, 2009; Greene and Loscalzo, 2017; Kanzian et al., 2019; Rinofner-Kreidl, 2020). These viewpoints pay more attention to the subjective and contextual experiences of the whole person (intrapersonal, interpersonal, and transpersonal); and include a more collectivist understanding of how human beings are interconnected with each other and their environment. Given that “survival” is the core aim of a hospital, it makes sense that healthcare systems have evolved in this way. That said, and in an age where there is a move to make healthcare more compassionate and person-centered, working in a holistic manner can be difficult because the system has emerged from a very different standpoint.

It is worth considering this in terms of Gilbert’s (2017) pioneering research on compassion and Compassion Focused Therapy (CFT). Gilbert (2017) views compassion through an evolutionary lens, as a motivational system rooted in mammalian caring. It is defined as: *“the sensitivity to suffering in self and others, with a commitment to try to alleviate and prevent it”* (Gilbert, 2017, p. 25). Gilbert (2005) outlines three emotion regulation systems that influence compassion. The threat system (detection and protection), the drive system (resource acquisition and achievement) and the soothing system (safeness, caring, contentment). Due to the demands of busy hospital settings, many healthcare workers are frequently oscillating between the threat and drive systems with little space for soothing. This is considered further below, from a neurobiological perspective, in terms of the influence of the vagus nerve on compassion.

The process outlined above influences how healthcare professionals care for patients; how staff members engage with each other; and how teams and departments interact. The system prioritizes certain aspects of individuals’ experiences whilst de-prioritizing others. Prioritized aspects include the physical disease of the patient; the problem-solving, conceptual and intellectual tasks of staff; and differences between patients and staff. The corollary of this is that the subjective, emotional and holistic experience of the patient can get marginalized; the emotional and human impact of this work on staff is under-acknowledged; and the interconnectedness of patients and staff is given less attention. What follows, focuses on a number of these issues within a cancer context.

This paper explores how the emotional distress experienced by patients impacts staff; an understanding of this process from a psychotherapeutic and neurobiological perspective; the influence of the wider hospital-based system within this; and working with this phenomenon within a team. Fictitious case studies are used to link the broader theoretical and conceptual learnings with the clinical and experiential aspects of working on the frontline. The perspectives informing the development of the paper are pluralistic, integrative and interdisciplinary (Norcross and Goldfried, 2005; Boix Mansilla, 2010; Teo, 2010). It combines learnings from a range of fields, including Psycho-Oncology (Watson and Kissane, 2011), Transference and Countertransference (Jacobs, 2017), Process Oriented Psychology (POP; Mindell, 1988, 2017), Group Psychotherapy (Yalom and Leszcz, 2005), Mirror Neuron Research (Rizzolatti and Craighero, 2004; Iacoboni, 2008), Poly Vagal Theory (Porges, 2011), Compassion Focused Therapy (CFT, Gilbert, 2017) and Systems and Field Theory (Lewin, 1951; Bertalanffy, 2015).

The integration of psychological knowledge from multiple perspectives and applied to a cancer context is one of the novel aspects of this paper. A further contribution is the awareness the paper brings to relationships, situations and dynamics that often get overlooked and consequently are harmful for patients and staff.

This process of taking psychological knowledge from different areas and assimilating it in a way that is relevant, meaningful and digestible to other practitioners is one of the key roles of a hospital-based psychologist. This is in keeping with the Scientist Practitioner Model (Baker et al., 2000) and being a Reflective Scientist Practitioner (Donati, 2016). The latter highlights the need for closing the mind-body gap while also integrating the more intrapersonal and transpersonal aspects of people’s experiences, and how these can exist in both an individual and interconnected way.

## The Emotional Impact on Patients and Staff

People who receive diagnoses and treatment for cancer face a vast range of challenges. They contend with the impact of treatment, physical pain and discomfort, procedural and hospital-related uncertainty and many other problems that arise when interfacing with a healthcare system. The effects of cancer and treatment can be profoundly destabilizing to core aspects of one’s identity, sense of physical integrity, meaning and place in the world (Yalom, 1980; Van Deurzen and Arnold-Baker, 2018) and the process of psychosocial reorganization can be non-linear and complex (Le Boutiller et al., 2019, 2021). Some aspects of this reorganization may remain unresolved, particularly if the person lacks a safe and supportive context (relational, financial, social) to process these changes. The combined effect of these issues results in cancer patients and their loved ones experiencing significant emotional distress (Watts et al., 2014, 2015; Yang et al., 2016; De Laurentis et al., 2019; Klein et al., 2019; Peng et al., 2019).

Healthcare practitioners working with this group of patients also suffer substantial distress. Compassion fatigue, burnout and occupational and vicarious stress are very prominent among people working in cancer care (Arimon-Pagès et al., 2019; Guo and Zheng, 2019; Todaro-Franceschi, 2019; Gribben and Semple, 2021). This paper considers one of the often unconscious dynamics that contributes to how emotional distress experienced by patients can affect staff.

### A Transferential Perspective

Box 1 outlines a scenario that is likely familiar to many frontline practitioners, and illustrates a quality of interpersonal effect that is formally termed “transference” (Jacobs, 2017). Derived from developments in Freud’s (1904) psychoanalytic ideas, transference describes an interaction where one person experiences in themselves the desires, thoughts, feelings and attitudes that another person is not consciously aware of, or expressing explicitly, yet is conveying implicitly through unconscious signals. Traditionally, countertransference was viewed as the therapist’s responses (e.g., thoughts, attitudes, emotions) to the client, particularly those that are further from consciousness (Freud, 1904). More recently, countertransference has been viewed from a more relational perspective, where the “transferring” can be initiated by either party. Gabbard (2001), views this process as mutual and co-created with both therapist and client contributing. The transference may be more likely to impact the therapist where it is an area of vulnerability for them.

**BOX 1. The emotional impact of working with patients in cancer care.**

***Serena and Hamish***

*Serena is a Clinical Nurse Specialist (CNS). On a Thursday morning, Serena goes to meet with Hamish, a 65 year old man, who is currently an inpatient. Upon entering the single room where Hamish is staying, the first thing Serena notices is the unusual quietness. She sees Hamish sitting on the bed and knows he’s there but it feels like something is missing. Serena says hello, at which point Hamish turns his head around. Serena is greeted with his gaunt, skeletal face. She makes eye contact with him and notices his shallow breathing and the downward motioning of his mouth and lips. On reflection, she notices how her “heart sank” at this moment and how a brief image of her own late father, who died a year previously, came to mind. She brushes it out of her mind so as to “be a good nurse” and “properly care for Hamish.” She tries to focus on Hamish, slowing her own breathing and giving more attention to Hamish, allowing silences where possible. Hamish tells Serena how he is afraid of dying. He speaks of being worried about leaving his wife and children behind and how they would manage, if he dies. Hamish has three daughters, aged 27, 25, and 23. He says that he worries in particular about his youngest daughter, Alice who has a moderate learning disability and who he and his wife have always cared for together. He also worries about the impact of this on his wife. Serena notices a tightening in her chest over the course of the conversation but notices it more strongly at this moment. She notices her own anxiety increasing but continues to take slow deep breathes to support herself. Serena’s middle daughter has a mild learning disability and has been having difficulties at school. This has been challenging for her and her husband. Serena feels some tears emerging in her eyes at this point but brushes them away. All the while a sensation on the left side of her chest becomes more and more pronounced. She described it as a “dull ache.” Serena left Hamish’s bedside that afternoon with that so called “dull ache” weighing her down, a “heavy heart” and a “foggy head.”*

From a POP perspective, there are six channels through which a person’s internal processes can be experienced and perceived by others (Mindell, 1988). This includes four irreducible channels (auditory, visual, proprioceptive, and movement) and two composite channels (relationship and collective/world) (Diamond and Jones, 2004; Cotter, 2021a). When a process is communicated via the proprioceptive channel, it is experienced in an embodied, “felt sense” manner, via bodily and physical sensations. Where a client cannot express emotional experiences in a proprioceptive channel (e.g., because it would be too overwhelming or anxiety provoking; or it does not feel safe enough to do so) those experiences may emerge in the therapist via the relationship channel (Goodbread, 1997).

In therapy, transference is significant and useful, and the role of the therapist is to manage this process through watching and reflecting on the client’s communication signals at all levels. The “reflective capacity” of the therapist has been found to have a significant bearing on the outcome of therapy (Gabbard, 2001; Hayes et al., 2011). This process has been largely understood within a client-therapist dyad, however, it also occurs outside of therapy within other relational dyads, groups and systems but people are often less aware of it (Hollwey and Brierly, 2014; Mindell, 2014; Cotter et al., 2017). An understanding of these processes, and capacity to facilitate them, can be useful at a collective and systemic level to support groups of people to work more effectively together. This is particularly true in emotionally distressing situations, where interpersonal and inter-team conflict are more likely. For instance, supporting a group of cancer nurses within one department to experience and process emotional difficulties in a safe and containing way can improve intra-team relating and communication. Facilitating their colleagues in a related department in a similar way can enhance inter-team relating between the two groups.

### A Neuroscientific Perspective

In keeping with the pluralistic and integrative nature of this paper, a neurobiological understanding of the transference process described in Box 1 is outlined below, as an additional and complementary standpoint to other phenomenological and psychodynamic perspectives. This viewpoint is grounded in the same positivist epistemology as the medical model (Playle, 1995). It is worth bringing the reader’s attention to this because difficulties arise when this is taken as *the only way* of determining knowledge. It is important to draw the distinction between “another perspective” and “proving” or “lending credibility to.” One of the harmful artifacts of the scientific method and often associated quantitative research is the notion that subjective experiences have to be “proven” or “verified” in some objective manner for them to be considered “true” or “acceptable.” This mindset often contributes to people’s subjective experiences being invalidated and undermined. Holding this awareness in mind, the reader is invited to consider two relevant areas of neurobiological research.

#### Mirror Neuron System

Mirror neurons were first observed at the start of the 1990’s (Rizzolatti et al., 1996) and while this field is continuously evolving it offers a number of points worth considering. These neurons appear to have an active role in the neural pathways that facilitate empathic responsiveness, through attuning and responding to facial expressions, postures and vocal inflections (Iacoboni, 2008). They do not operate in isolation but form a neurological component of broader social and emotional intelligence functions (Rizzolatti and Craighero, 2004). Some theorists suggest that it is more accurate to use the term “Mirror Neuron System” (MNS) as it is not possible to be precise about mirror neurons in human beings (relative to animals in animal studies).

According to Gallese et al. (2007), the activity of the MNS underpins the capability of an observing individual to simulate the emotions of another person in an automatic, unconscious and non-inferential manner. This body-related experiential knowledge allows the observer to grasp or sense the kinds of emotions the observer may be experiencing without cognitive or language processing and typically occurring outside of consciousness (Siegel, 2006). This system enables people to “feel into” what it would be like if they were in a situation that they observe another person in, as opposed to more conscious mentalizing, rationalizing or thinking about it. It is not that one person’s feeling state is mirrored exactly but rather the observer mirrors what they would feel themselves if they were in the other person’s position. Some theorists suggest that “attunement” or “congruent responding” would be more accurate than the term “mirroring” (Gallese et al., 2007). It is the interaction between the observer and the emotional state of the observed that is most important. This is why a practitioner may become emotionally affected by one patient but not another. In short, the MNS is not free of personal and cultural bias but is heavily influenced by them (Keestra, 2012).

The counterpoint view is that the MNS is only one aspect of complex and multifaceted phenomena, such as empathy or social and emotional intelligence (Alford, 2016). Similarly, psychoanalysts caution against reducing “mind” to “brain” and endeavoring to explain complex processes like transference and countertransference from a reductionist perspective (Vivona, 2009). Recent review studies indicate that there is direct evidence for a relationship between mirror neuron activity and empathy, however, lots of questions remain regarding mirror neurons in human beings (Hyeonjin and Lee, 2018; Bekkali et al., 2020).

#### Polyvagal Theory

Historically, the autonomic nervous system has been divided into sympathetic (“fight or flight”) and parasympathetic (“freeze”) components (Ogden and Fisher, 2014). Porges’ (1995, 2011) Poly Vagal Theory (PVT) further subdivides the parasympathetic system into two distinct elements, giving three different sub-systems. These are hierarchically organized in terms of level of arousal relative to level of threat.

The first subsystem, which is connected to the lowest level of arousal, is concerned with social engagement. It regulates areas of the body used for social and environmental interaction (e.g., facial muscle expression, eye gaze, tone of voice). It is often called the “Social Engagement System” and is the most evolutionary recent and sophisticated of the three subsystems. It is facilitated by the ventral (front) branch of the vagus nerve and is the first subdivision of the parasympathetic nervous system. When a person’s level of arousal remains within a “window of tolerance” they have the capacity to interact and engage with people in a social manner. In non-threatening environments, this system helps people to form social bonds and sustain intimate relationships.

When a person becomes hyperaroused because of a threat in their (internal or external) environment the amygdala activates the hypothalamus. This activates the sympathetic system and the individual is prepared for mobilization (fight or flight). This is the second of the three subsystems. By this point they are much less capable of social engagement. The third and phylogenetically older subsystem involves the parasympathetic system again and in particular the dorsal (back) branch of the vagus nerve. This occurs when someone becomes hypoaroused and the level of threat leads to immobilization or a freeze response. Albeit an oversimplification, the ventral vagus acts as a vagal brake—inhibiting and disinhibiting sympathetic defense-oriented fight or flight behaviors whereas sensitive modulation of the vagal brake promotes social affiliative behaviors and interactions (Fiskum, 2019).

These findings have been deployed within the field of sensorimotor psychotherapy and related approaches, especially in working with trauma (Ogden and Fisher, 2014; Ogden, 2021). From a PVT perspective, neuroception is a term used to describe the automatic capacity of the nervous system to evaluate safety and risk in one’s environment without conscious awareness (Flores and Porges, 2017). Deep connection or attunement between therapist and client can activate the client’s social engagement system reducing defenses and invoking a neuroception of safety. Emotion Focused Therapy (EFT) has conceptualized this as “therapeutic presence” (Geller, 2018). Emotion regulation is promoted in relationship through right brain to right brain communication (Quillman, 2012; Schore, 2012). The wider a therapist’s window of tolerance the greater capacity they have to maintain social engagement in very anxiety provoking situations (Flores and Porges, 2017). Remaining compassionate in this way, toward another person in distress, appears to be positively linked with vagally-mediated heart rate variability (HRV; Di Bello et al., 2020).

### An Integrative Perspective

It is useful to reconsider the situation described in Box 1 in light of these different perspectives. Hamish tells Serena his story and unconsciously “transfers” emotional distress onto her. This activates emotional experiences in Serena that are triggered by different parts of Hamish’s transference. In particular, Serena is activated by the parts of Hamish’s story that resemble her own. This results in a countertransference response from Serena.

Serena experiences a lot of emotion because her mirror neurons produce an emotional response based on what she expects she would feel if in Hamish’s position, which in this case has been very close to experiences she has had in her own life. This may explain the intensity of the feelings that Serena experienced.

Serena experiences a lot of anxiety and emotionality more broadly in response to Hamish. However, she is able to sooth and regulate this distress. Consequently, Serena sustains her own level of arousal within a window of tolerance, remains socially engaged and attuned, and sustains a safe space for Hamish to express himself—invoking a neuroception of safety. This occurred with limited awareness from either party. Had Serena’s level of arousal reached an intolerable level or she remained unaware of Hamish’s distress the outcome is likely to have been different.

## Systemic Thinking and Collective Working

Cotter et al. (2019) have previously discussed how frontline practitioners can use different practices to stay present to and support patients during distressing situations. These include focusing on the present moment with patients (“mindful engagement”); punctuating the day with mindful pausing; being present to one’s own behavior, body, emotions and thoughts; moving toward (rather than away from) patient interactions that evoke difficult emotions; and not only “doing” but also “being with” patients. As well as facilitating dignified and holistic care for patients, these practices support practitioners’ coping at an individual level, which can have further positive knock-on effects for patients. From a systemic perspective, staff-support is patient-support (Cotter et al., 2020).

This paper builds on this earlier work and focuses on how practitioners can be further supported to cope, and how team working can be a chief component of this. Box 2 describes three scenarios that practitioners might find themselves in while trying to cope with the situation outlined in Box 1. Different areas of study are used to develop a greater understanding of group processing within these situations and how it can be helpful or unhelpful.

**BOX 2. Collective processing of distressing emotions within a team.**

***Serena and her Team***

*Scenario I —Systemic Blanking:*

*From past experiences, Serena knows that there is no point in discussing her experience with her team. She experiences her colleagues, David (Team Lead) and Maggie, who have been in the team the longest, as being “cut-off” and knows that they would look down on her for feeling “vulnerable.” Another colleague, Sue-Lyn feels like she cannot engage in such a conversation because of how it would be perceived by David and Maggie. David and Maggie have learned over time to avoid such conversations. Their previous Team Lead, Alberta was an “old school nurse,” who mocked people when emotional experiences were mentioned. Alberta is David’s supervisor. He continues to have to negotiate this part of Alberta’s personality. Alberta grew up in Apartheid South Africa and “cutting off” from emotions was a very important way of surviving. This coping mechanism has been valued within her nursing career where she has always been reinforced for her professional, organizational and “just get on with it” skills.*

*Scenario II—Collective or Team Blanking:*

*On returning to the office, Serena goes into a meeting with Sue-Lyn, Maggie and David. Serena struggles with answering the team’s questions and tuning into the meeting. David, who is chairing the meeting, gets annoyed with Serena and highlights how much they have to get through. Serena tries to concentrate but feels worse following David’s comment. Later in the morning, Serena tries to discuss the issue with Maggie. Maggie alludes to how busy she is and Serena decides not to say anything. By lunchtime, Serena is still feeling overwhelmed. She decides to share her situation with Sue-Lyn and Maggie. Sue-Lyn notices that David is listening in the background and feels like she has to conduct the interaction in a particular way (effective, productive, intellectual). She focuses on the procedural and clinical issues and doesn’t acknowledge Serena’s feelings. Sue-Lyn, who is new to the team, knows that David would look down on anything else. Maggie, who is not comfortable with a more emotion-focused conversation, doodles on a piece of paper and sends a message on her phone. Serena feels very alone by the end of the day; she feels like it was her fault for not being able to cope; she feels like she is not able for the work; and should have handled the situation better. She calls in sick for the rest of the week and “feels off” for a number of weeks thereafter.*

*Scenario III—Collective Processing:*

*Serena goes back to the office and sits at her computer. She finds it hard to concentrate. Her colleague Sally notices that something seems “a little off.” She asks if everything is okay but Serena says everything is fine. Just after lunch Serena sits back at her desk where Sally, Maya and Daniel are chatting. Sally asks Serena if everything is okay, at which point, Serena bursts into tears. As she does, Sally, Maya and Daniel let go of what they are doing and turn around to Serena. They give Serena some time and space, attune to her eye contact and facial expression, slow themselves down, ask open questions about feelings and the situation with Hamish. They avoid advising or problem-solving. Serena tells them about Hamish and how it connects with her own life and the difficulties she and her family are experiencing. The team checks in on Serena the next day, later in the week and agree to come back to discuss it further again in their facilitated Level 2 supervision session, as it has raises issues for many team members.*

From a Systemic and POP perspective (Bertalanffy, 2015; Mindell, 2017), hospital-based systems often centralize certain ways of working, communicating and interacting. For instance, problem-focused coping can be more central than emotion-focused or relationship-focused coping (Lim et al., 2010; Labrague et al., 2017). The individual positions of patients and staff are more dominant than recognition of the degree to which people within the system are interconnected. The impact of people on each other is greater than is often acknowledged; and as a whole the influence of the overall system on the people within it is often overlooked.

This dynamic contributes to the kind of “Systemic Blanking” of Serena’s emotional distress outlined in Scenario I. This culture may be evident across the overall system (e.g., hospital), sub-systems within it (e.g., individual departments), groups within sub-systems (e.g., cancer CNSs), and individual relationships (e.g., patients and staff or staff and staff). Where such a culture dominates, it results in practitioners feeling unable to view their emotional experiences as needing time, attention and care. The kind of “Collective or Team Blanking” described in Scenario II is an example of this phenomenon occurring at a more localized level. For a range of systemic and personal reasons, and the interaction between them, the team does not attune to, or engage with, Serena’s emotional experiences. They focus on problem and solution-focused coping, as opposed to a more emotion-focused approach within relationship.

In contrast to scenarios I and II, scenario III represents a situation where collective processing is valued and integrated into regular daily working. MNS (Gallese et al., 2007) and PVT (Flores and Porges, 2017) research with people in groups highlights some of the neurobiological mechanisms through which an emotionally understanding team can be supportive. Where a practitioner is faced with a group of colleagues who are attuned, mindful and present to their emotional distress it can be very relieving. When each member of the team connects via their MNS and remains socially engaged via the ventral vagus nerve it creates an environment in which the distressed practitioner can process their emotions and relieve themselves of the emotions they have had “transferred” on to them. Bubler (1958) famously spoke of the “I—Thou” encounter that becomes possible when two people become present with each other. A group of practitioners supporting each other might be considered an “I—Thee” encounter. This cannot be separated from the wider system and creating an environment in which people feel safe enough to be open with their emotional vulnerability is key.

Some teams ably operate in this way. This occurs where the number of people within a team who are more emotion-and-relational-focused surpasses a threshold, changing the prevailing culture, or rather altering it slightly. This can be especially true where a team leader supports this way of working and models it for the team, much in the way that compassionate leadership is described by de Zulueta (2016) and West (2021). Where the person with the greatest positional or contextual power (Mindell, 2014) supports a more marginalized way of working, it can be easier to integrate it. This is also the case where unofficial leaders or “influencers”—people with high social or psychological rank (Mindell, 1995)—support this way of working and relating.

Where a more problem-focused culture (in isolation) prevails, teams are likely to struggle with this part of their work. This is an inevitability where groups of people have to contend with significant amounts of emotional distress on a daily basis without any dedicated means of processing this emotion. Extrapolating from the examples provided in scenarios I and II, it is easy to imagine how collective or team blanking may result in a cumulative build-up of stress over time and feelings of inadequacy amongst employees. Without allowing time and space for team members to understand each other’s perspective and emotional experiences, team members can become critical of each other and interact in interpersonally ineffective ways. For example, feelings of inadequacy or difficulty processing emotions may be projected onto other colleagues, leading some practitioners to take a very critical, expert stance. For others, difficult emotions may be repressed, leading to significant anxiety and a sense of threat or emotional “shut down” within the work context.

The dynamics that promote or inhibit this kind of culture are often partly unconscious and to a degree unspoken about. The further a team is from this way of working or the further influential people within the team are from encouraging it, the more unconscious and unspoken about it may be. This makes it difficult to work with and bringing an awareness to it requires timeliness and caution. There are often historical individual (e.g., Alberta growing up in Apartheid South Africa) and group (e.g., David and Maggie doing their best to cope for many years) issues that have given rise to the situation evolving as it has. Marginalizing emotions in this way is often a necessary way of coping and there may be much hurt that has given rise to it. In short, marginalizing the emotional experiences of staff impacts team cohesiveness and capacity for team working, in turn affecting the capacity for collective processing. Understanding and facilitating group process can take time, but is ultimately supportive of better team functioning.

## The Impact of Individualistic Working

This final section considers how a culture that fails to acknowledge the impact of patients’ distress on staff, and leaves practitioners isolated in trying to cope, can have a negative impact on both healthcare workers and patients. Box 3 outlines three scenarios that reflect the types of interactions that can emerge when this type of working is under acknowledged. These scenarios are presented individually for clarity but it is likely that versions of all three happen together and in an unpredictable manner.

**BOX 3. Lone coping with distressing emotions.**

***Hamish on his Own***

*Scenario 1—Unconscious busy-ness:*

*Tim, a physiotherapist has a busy schedule and is under pressure because a colleague has called in sick. When speaking with Hamish he is thinking about Hamish and his needs while also thinking about the other patients and tasks he has to do. Tim does not notice the tone of Hamish’s voice, the long pauses between his words, his downward motioning facial expression, and his general apathy. Tim is happy to get through the meeting and on to the next task. Hamish is left feeling alone.*

*Scenario II—Self-preservation:*

*Mary, an occupational therapist is looking forward to a dinner-party she is going to tonight—its 3 p.m. on a Friday. Mary has to check in with Hamish to ascertain how he is progressing with the program she has set. She has previously felt overwhelmed following exchanges with Hamish and somewhere at the back of her mind she is aware that she does not want that to happen this afternoon. Mary “shuts down” in an emotional sense. She focuses her assessment in a very linear (e.g., sticks to her task-list), procedural (e.g., focuses on the OT intervention), professional (e.g., avoids discussing how Hamish is feeling or engaging more personally herself) manner so as to get through the session without opening up “any can of worms.” Hamish is left feeling alone.*

*Scenario III—Anxiety Response takes over:*

*Serra, a junior doctor goes to see Hamish. She hasn’t had time to read much of the notes and rushes from a meeting to go see him before a scan. As soon as she walks in the door, she notices Hamish sobbing. He starts to tell her his story and her heart rate increases, she feels overwhelmed and finds it hard to communicate with Hamish. She notices her head goes “fuzzy”; feels an “achey pain in her chest”; and her stomach is “a bit off.” Serra is relieved when a nurse interrupts and says that Hamish has to go for his scan. Hamish is left feeling alone.*

In the first scenario, the pressure that Tim is under and consequent busy-ness does not allow him to be present to Hamish and attune to his verbal or non-verbal behaviors. In scenario II, Mary’s past experience of being emotionally distressed following encounters with Hamish (and not being able to process these experiences) gives rise to an unconscious anxiety that results in her surpassing her window of tolerance. As a result, she doesn’t socially or emotionally engage with Hamish. A similar process occurs to Serra in scenario III but in an even more unconscious and instantaneous manner. In each of the latter situations, the ventral vagus nerve is overtaken by the anxiety response. In all situations the consequence is Hamish feeling alone.

This loneliness or isolation may occur at a number of different levels. Interpersonal isolation arises when a person feels lonely in the presence of other people and is most obvious in the situations outlined. However, this interpersonal isolation can have further knock on effects on patients with cancer diagnoses, especially for those who fear their own death or are actively nearing end-of-life. Intrapersonal and transpersonal isolation arises where someone becomes disconnected from parts of the self or a sense of something beyond the self, respectively—the latter may vary significantly, depending on a person’s belief system (Kearney, 1996; Shaver, 2002). Existential isolation represents the separation between the individual and all the other individuals in the world (Yalom, 1980). It represents the extent to which humans come into this world alone and leave it alone. Mindful, present-focused interpersonal engagement and interaction from healthcare professionals can support patients in coping with these different manifestations of loneliness (Chochinov and McKeen, 2011; Cotter et al., 2019).

As long as practitioners have to contend with significant levels of emotional distress in their patients and themselves, without having appropriate avenues for processing and coping, this can contribute to complex team conflicts and impact people’s lives outside of work (Rosen et al., 2019; Maglalang et al., 2021). If patient-facing work requires one to regularly “shut down,” as a means of protecting oneself or coping, this “shutting down” may affect other personal relationships. When this pressure interacts and combines with current healthcare workplace pressures (e.g., resource constraints, excessive expectations, workforce shortages) it is likely to be contributing to levels of compassion fatigue, burnout and job satisfaction becoming ever more problematic (Cocker and Joss, 2016; Lu et al., 2019; Shi et al., 2022), especially since the onset of COVID-19 (Xu and Zhang, 2020).

## Discussion

As a prelude to considering implications for practice and future research, it is important to understand the phenomenon outlined throughout—and not just intellectually but also at an emotional and relational level. It is important that we do not move too quickly to: *“What can we do? What can we change? And how can we solve the problem?”* This is the very mindframe that requires changing in the first instance. If a change of perspective is not introduced as a starting point, all efforts thereafter will likely be ineffective or at least much less effective. This is often more complex, requiring greater awareness, than making external alterations at structural or policy level and often why it is the step that is overlooked.

Taking the above as a necessary starting point, there is potentially much to be gained for staff and patients if the kind of collective processing outlined in Scenario III can be engendered within frontline teams. Much has been written about the impact of a supportive group setting in helping people to process emotional and relational difficulties. Group psychotherapists have been doing this across a range of settings and populations for many years (Yalom and Leszcz, 2005). POP groups have flourished in particular in post-conflict settings (Audergon et al., 2010; Audergon and Audergon, 2017). Group-based approaches may be especially important where emotional distress occurs within group-oriented or systemic situations in the first place (Reiss, 2018). For instance, Psychodynamic-oriented groups have been used with frontline NHS staff working in mental health settings (e.g., Thorndycraft and McCabe, 2008).

In the daily practices of hospital teams, forms of collective processing can be woven into procedural, organizational, task and problem-focused working. This is best conceived of as a way of working and facilitating group discussion as opposed to some separate or additional intervention. It may also be introduced from different existing models or approaches. For example, from a mindfulness perspective (Kabat-Zinn, 2018), this would involve a team being mindfully present to the practitioner holding the distress (Cotter et al., 2019), whereas from a narrative therapy orientation (Payne, 2014), it would involve the practitioner retelling their story with the support of their listening colleagues.

One specific example of a group-based collective processing approach that has been used with frontline healthcare practitioners is *staffSPACE (Stopping to Process and Consider Events)* (Cotter, 2021b). This is currently being adapted for use in cancer care and is being trialed as a pilot project by the authors. This will be explored initially from a qualitative perspective and later evaluated from a more quantitative standpoint (Creswell and Creswell, 2018). A mixed-methods approach will be used as part of this work. Schwartz Rounds (Robert et al., 2017) are a further group-based approach that involve collective processing. They have been evaluated using a realist informed mixed-methods approach and shown to offer distinctive support for healthcare workers and positively impact staff wellbeing, empathy and compassion for colleagues and patients (Maben et al., 2018).

The issues raised throughout are also relevant at a broader level. “Interdisciplinary” working (Boix Mansilla, 2010) is viewed as being very important within modern systems, however, it is generally recognized that it has not been as effective as was hoped (MacLeod, 2018). More has to be done to support people within hospitals to work together, especially given the high levels of stress, limited resources and heterogeneous patient and staff groups. Process-oriented and systemic facilitation, thinking and planning around complex dynamics between patients and staff, within teams and between departments, has a lot to offer. Hospital-based systems would do well to develop departments focused on supporting team working that sit alongside individual medical specialities, in the same way as do teams responsible for infection control or information governance. It is important that support for collective working is given a place in its own right—as a specialism in itself—within the hospital-based system. If there is to be truly holistic and integrative care, there needs to be an entity within the system whose primary role is devoted to this. This process is likely to be ever changing and evolving, as well as necessary in order for systems and sub-systems to work in a more cohesive manner.

In sum, there is ever growing recognition of the need for healthcare to supplement the gains it has made in improving survival with more integrative, holistic and systemic working. Social medicine, network medicine, systems medicine and systems biology have much to offer in this regard (Ahn et al., 2006; Miles, 2009; Federoff and Gostin, 2017). These sub-fields are focused on creating “a medicine of the whole person” and “putting the patient back together” (Miles, 2009; Greene and Loscalzo, 2017). The authors are of the viewpoint that one step further is needed. In addition, to putting the patient back together it is also necessary to put the whole hospital back together. The future of hospital-based healthcare lies in not only working with the whole patient but the whole hospital system, all of the people within it and the interactions between them.

## Author Contributions

This manuscript was based on practice-based working within a public health service psycho-oncology department. It involved regular consultation and discussion between all authors. PC drafted the manuscript, was responsible for conceptualizing the manuscript and was the first author. AH, SN, and CJ participated in consultations regarding the content of the manuscript, reviewed the manuscript, and provided feedback on it. CU provided overview of manuscript. AK participated in consultations, reviewed the manuscript, provided feedback, and was the supervising author. All authors contributed to the critical review and approved the final version of the manuscript.

## Conflict of Interest

The authors declare that the research was conducted in the absence of any commercial or financial relationships that could be construed as a potential conflict of interest.

## Publisher’s Note

All claims expressed in this article are solely those of the authors and do not necessarily represent those of their affiliated organizations, or those of the publisher, the editors and the reviewers. Any product that may be evaluated in this article, or claim that may be made by its manufacturer, is not guaranteed or endorsed by the publisher.
